# Individual heterogeneity in reproductive rates and cost of reproduction in a long-lived vertebrate

**DOI:** 10.1002/ece3.615

**Published:** 2013-05-31

**Authors:** Thierry Chambert, Jay J Rotella, Megan D Higgs, Robert A Garrott

**Affiliations:** 1 Department of Ecology, Montana State UniversityBozeman, Montana, 59717; 2 Department of Mathematical Sciences, Montana State UniversityBozeman, Montana, 59717

**Keywords:** Bayesian statistics, individual variation, life-history theory, marine mammals, population dynamics, posterior predictive checks

## Abstract

Individual variation in reproductive success is a key feature of evolution, but also has important implications for predicting population responses to variable environments. Although such individual variation in reproductive outcomes has been reported in numerous studies, most analyses to date have not considered whether these realized differences were due to latent individual heterogeneity in reproduction or merely random chance causing different outcomes among like individuals. Furthermore, latent heterogeneity in fitness components might be expressed differently in contrasted environmental conditions, an issue that has only rarely been investigated. Here, we assessed (i) the potential existence of latent individual heterogeneity and (ii) the nature of its expression (fixed vs. variable) in a population of female Weddell seals (*Leptonychotes weddellii*), using a hierarchical modeling approach on a 30-year mark–recapture data set consisting of 954 individual encounter histories. We found strong support for the existence of latent individual heterogeneity in the population, with “robust” individuals expected to produce twice as many pups as “frail” individuals. Moreover, the expression of individual heterogeneity appeared consistent, with only mild evidence that it might be amplified when environmental conditions are severe. Finally, the explicit modeling of individual heterogeneity allowed us to detect a substantial cost of reproduction that was not evidenced when the heterogeneity was ignored.

## Introduction

Differences in the reproductive and survival abilities of individuals in a population have long been considered a key feature of life (Darwin 1859). The investigation of the prevalence, nature, and underlying mechanisms of such individual heterogeneity in fitness components is relevant to both evolutionary ecology (Wilson and Nussey 2010; Bergeron et al. 2011) and population dynamics (Lomnicki 1978; Kendall et al. 2011). First, interindividual variability associated with fitness differences is, with heritability, one of the fundamental principles of the theory of natural selection (Darwin 1859; Endler 1986). Furthermore, we know that the detection *in natura* of reproductive trade-offs and senescence patterns, expected from life-history theory, can be hampered when consistent individual differences are ignored in demographic studies (Vaupel and Yashin 1985; Van Noordwijk and De Jong 1986; Service 2000; Cam et al. 2002; Nussey et al. 2008; Weladji et al. 2008). Finally, it has been shown that heterogeneity in vital rates could change the magnitude of demographic stochasticity and thus affect population dynamics and persistence (Lomnicki 1978; Conner and White 1999; Vindenes et al. 2008; Kendall et al. 2011). In terms of mechanisms, individual heterogeneity has been related to genetic characteristics (Foerster et al. 2003; Hunt et al. 2004), as well as to innate and acquired phenotypic traits, such as physical features (e.g., Andersson 1989; Zuk et al. 1990; Festa-Bianchet et al. 1997; Bérubé et al. 1999; Vanpé et al. 2007), physiology (Burton et al. 2011; Pryke et al. 2012), and behavior (Dall et al. 2004; Smith and Blumstein 2008). Alternatively, individual heterogeneity may also be explained by variations in conditions and resources during early development (Lindström 1999; Festa-Bianchet et al. 2000; Metcalfe and Monaghan 2001; Lummaa and Clutton-Brock 2002; Hamel et al. 2009b), in environmental conditions throughout life (Landis et al. 2005), in parental care (Hunt and Simmons 2000; Schwagmeyer and Mock 2008), and in other maternal effects (Mousseau and Fox 1998; Fox et al. 2006).

Although among-individual variability in fitness is generally assumed to be ubiquitous in nature (Bergeron et al. 2011; Kendall et al. 2011), the extent to which it is actually prevalent in wild populations remains poorly understood, as evidenced by recent attention to the topic in the literature (Cam and Monnat 2000; Cam et al. 2002, 2012; Aubry et al. 2009, 2011; Hamel et al. 2009a,b, 2012a; Tuljapurkar et al. 2009; Steiner et al. 2010; Orzack et al. 2011; Plard et al. 2012). Individual heterogeneity in wild populations of vertebrates was first conceived and investigated through the “fixed heterogeneity” hypothesis (Cam et al. 2002; Bergeron et al. 2011). The two key features of this hypothesis are that (i) differences among individuals in performance are linked to some latent individual characteristics, and (ii) these latent differences are expressed in a constant (fixed) fashion over individuals’ lifetimes (i.e., across ages and the various environmental conditions experienced). The importance of the first of these features, that is, the existence of underlying differences in vital rates among individuals, has recently been questioned by the formulation of an alternative “dynamic heterogeneity” hypothesis (Tuljapurkar et al. 2009; Steiner et al. 2010; Orzack et al. 2011). This hypothesis recognizes that an individual's breeding and survival status can change (be dynamic) over a lifetime due to random chance alone. Thus, individuals with the same underlying vital rates can realize different life-history trajectory outcomes and underlying heterogeneity in individual vital rates need not be invoked to explain variability in realized fitness, unless observed levels of variation exceed those that can be generated by random chance and other sources of variation in individual vital rates and lifetime success, such as environmental conditions, individual's age, prior breeding experience, and current reproductive status. The relevant question with regard to the existence of latent heterogeneity in fitness components is thus: after accounting for these sources of variation, can the residual variability in realized fitness be explained solely by random chance or is there evidence for additional, unobserved, and perhaps unaccountable for sources of variation tied to individuals?

The second key feature of the “fixed heterogeneity” hypothesis could also be questioned, and an alternative view is that the expression of the underlying heterogeneity is variable, rather than fixed, over time. Indeed, individuals might differ in their ability to survive and reproduce, but such differences might not be consistently expressed across ages or environmental conditions (Lomnicki 1978; Hamel et al. 2009a,b; Lailvaux and Kasumovic 2011). This feature of individual heterogeneity in fitness components is especially relevant to the theory of natural selection, as the relative success of alternative phenotypes or genotypes also depends on the environmental conditions experienced (e.g., Van Noordwijk 1989). Such interaction between environment and latent heterogeneity could have two consequences: (1) the hierarchy of individuals, in terms of robustness, could change with environmental conditions, or (2) the hierarchy could be maintained, but the expression of underlying individual heterogeneity could be amplified in certain conditions (Lomnicki 1978), such that the amount of realized heterogeneity might vary across contrasted environments (Hamel et al. 2009a,b; Lescroël et al. 2009). For instance, it has been suggested that individual differences could be primarily, or only, expressed in unfavorable and stressful situations (Barbraud and Weimerskirch 2005; Tavecchia et al. 2005), whereas in favorable conditions, individuals would all perform similarly well. This idea is also supported by the fact that the strength of selective pressures at play in wild populations is generally quite variable over time (Siepielski et al. 2009) and seems to be dominated by rare episodes of very strong selection (Gould and Eldredge 1993).

**Figure 1:**
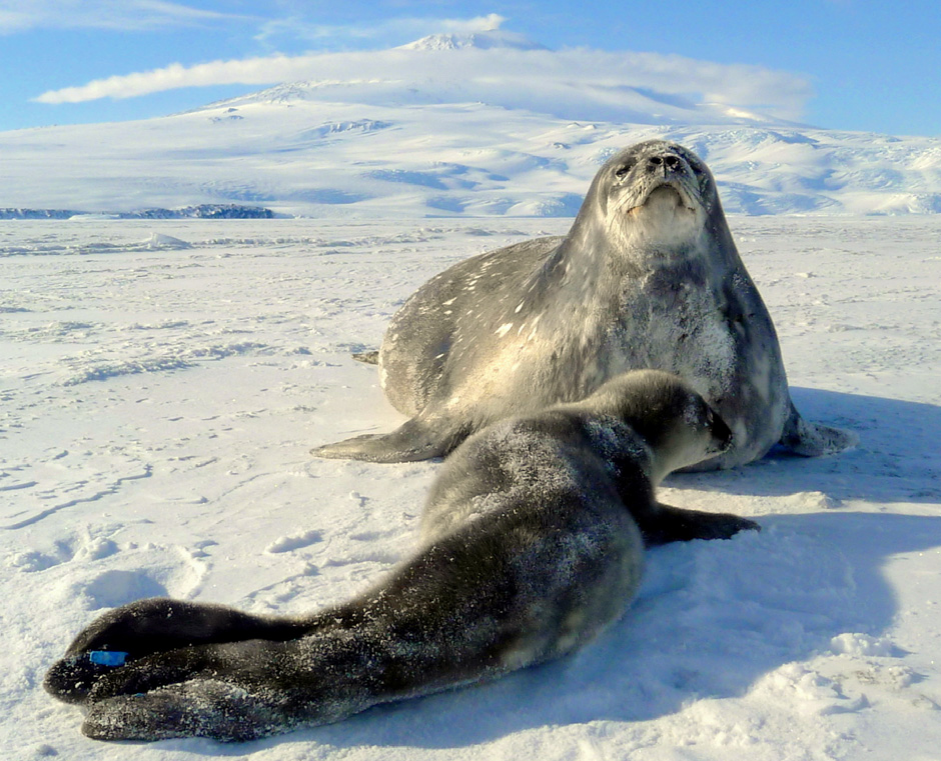
Female Weddell seal (*Leptonychotes Weddellii*) with her pup, in Erebus Bay.

In this study, using 30 years of mark–recapture data from a population of female Weddell seals (*Leptonychotes weddellii*, Fig. 1), we investigated (i) the relative support for the existence of unidentified (latent) sources of IH in reproductive rates, and (ii) the nature (fixed vs. variable) of the expression of underlying sources of variation across contrasting environmental conditions. Although as suggested elsewhere (Cam et al. 2002), individual heterogeneity in fitness components likely concerns both reproduction and survival, here we focus on reproductive rates because we expect them to display more heterogeneity given that, at the population level, they vary much more than do adult survival rates, which are very high and stable (Rotella et al. 2012). In these analyses, we focused on females that recruited to the breeding part of the population and did not include adult females that never produced a pup. Estimates of individual heterogeneity presented here thus pertain to the breeding portion of the population and are therefore conservative (i.e., represent minimum levels of heterogeneity in the whole population). To investigate the two questions of interest, the following three competing hypotheses were confronted: (H1) “no individual heterogeneity”: there is no substantial difference in reproduction probabilities among individuals; (H2) “fixed individual heterogeneity”: there is substantial individual heterogeneity in reproduction probabilities, and it is consistently expressed across contrasted environmental conditions; and (H3) “variable individual heterogeneity”: there is substantial individual heterogeneity in reproduction probabilities, and its expression varies with environmental conditions. Hypothesis H1 corresponds to a “null” model, as defined by Tuljapurkar et al. (2009), in which the observed differences in individual reproductive outputs are solely the result of the probabilistic nature of individual state transitions (“dynamic heterogeneity” hypothesis), whereas H2 corresponds to the “fixed heterogeneity” hypothesis (Cam et al. 2002). In H3, which corresponds to an additional alternative hypothesis (Hamel et al. 2009a,b), we contrasted the level of individual heterogeneity in “normal years” with that in a “perturbed period” of years when massive icebergs were present in the vicinity of seal colonies (2001–2005). This unusual perturbing event represented an episode of unfavorable conditions for the seals as evidenced by reduced reproductive rates (Chambert et al. 2012).

## Material and Methods

### Study population and data collection

The population of Weddell seals breeding in Erebus bay (Southwestern Ross Sea, Antarctica [77.62°–77.87°S, 166.3°–167.0°E]) has been the subject of a mark–recapture program since 1971 (Siniff et al. 1977), and since 1982, every pup born in the study area has been systematically marked shortly after birth. Each year, during the pupping season (October–November), seal colonies were visited every 2–3 days to tag newborn pups and untagged mothers, and five to eight surveys were conducted throughout the entire study area. During surveys, every encountered animal was recorded along with its sex and reproductive status. Animal handling involved in the collection of these data followed a protocol that was approved by Montana State University's Animal Care and Use Committee (Protocol #2011–38).

In this study, we used data from 1982 through 2011 to build encounter histories of individually marked females that were (i) of known age (i.e., tagged as pups inside the study area), (ii) part of the breeding population (i.e., females that had bred at least once), and (iii) resighted at least once after their year of first reproduction (recruitment) to provide information on reproductive rates (here defined as the “probability of producing a pup subsequent to recruitment”). The detection rate of mother–pup pairs is virtually 1.0 on the ice (Hadley et al. 2006), such that every year all females giving birth in Erebus Bay are detected. Moreover, female Weddell seals display strong philopatry, making it extremely rare for a female recruited inside the study area to later reproduce outside the study area (Cameron et al. 2007; Hadley et al. 2007a). Here, we restricted our analyses to data collected on females born inside Erebus Bay and known to have given birth there. Thus, we could reasonably assume that all reproductive events of this subpopulation of seals were recorded in our data, and any female not seen in a given year could be assumed to have skipped reproduction that year.

### Statistical modeling

Encounter histories started at the first reproductive event (state F) of an individual and thereafter consisted of two possible states: experienced breeder (E; as opposed to “first-time breeder F”) and skip-breeder (S). Probabilities of reproduction were defined as rates of transition from any state *k* (F, S, or E), in year *t*, into state E in year *t* + 1 (*ψ*^*kE*^). The complementary transition rates into state S corresponded to probabilities of skipping reproduction (1 − *ψ*^*kE*^). Based on previous analyses and knowledge of this population of seals (Hadley et al. 2007a; Rotella et al. 2009; Chambert et al. 2012), we modeled *ψ*^*kE*^ as a function of reproductive state in year *t* − 1 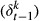 and year *t* (*η*_*t*_). We also included the standardized age of individual *i* in year *t* (*A*_*i*, *t*_) as a covariate in our models, but given that this study did not focus on age effects, our primary goal in doing so was to account for the potential confounding effect of age when considering our competing hypotheses about individual heterogeneity. We thus decided to model the age effect as a quadratic relationship because of its generality and biological relevance, and did not test alternative simpler age effects (e.g., linear trend, no trend). In order to investigate our three a priori hypotheses (H1, H2, and H3), we considered the influence of two types of individual random effects: (i) a “baseline” individual effect (*α*_*i*_) expressed in “normal” environmental conditions; and (ii) an individual effect (*β*_*i*_) expressed in “iceberg” years. Accordingly, we built a set of three competing models:


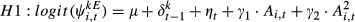



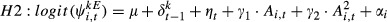



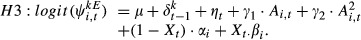


Here, *μ* represents a theoretical mean reproductive rate (on the logit scale), *γ*_1_, *γ*_2_ are the two parameters of the quadratic age effect, and *X*_*t*_ is a binary covariate indicating whether year *t* was an iceberg year (*X*_*t*_ = 1) or not (*X*_*t*_ = 0). We also note that in model H2, *α*_*i*_ corresponds to a unique random intercept for each individual *i* that is expressed in all years in keeping with the fixed heterogeneity hypothesis. On the other hand, in model H3, *α*_*i*_ represents the individual effect in noniceberg years (i.e., “normal” years), and *β*_*i*_ corresponds to the individual effect expressed during iceberg years (i.e., “disturbed” years).

In the analyses presented here, modeling was focused on the sequence of reproductive states during the time an individual was known to have been alive, that is, between its first reproductive event (state F) and its last encounter (in state E or S), a period we refer to here as the “minimal lifetime window” (MinLifeWin) of the animal. Models were thus conditional on the first and the last detection of each individual and did not include a survival parameter. This approach was sensible for our objective of evaluating possible differences among individuals in their frequency of reproduction, and not in their survival rates. Given the very high detection rates inside colonies (Hadley et al. 2007a; Rotella et al. 2009; Chambert et al. 2012) and the high philopatry of locally born animals in colonies (Cameron and Siniff 2004; Cameron et al. 2007), estimates of reproductive rates appeared to be very robust to this right censoring of encounter histories. Indeed, when we performed analyses (not presented in this study) using non-right–censored data in which survival and detection were explicitly modeled, we found that estimates of model parameters were the same. For ease of interpretation, we thus decided to present the results of the simpler approach focusing solely on reproductive rates. Furthermore, as noted before, any nondetection event inside an individual's MinLifeWin necessarily corresponded to the skip-breeding state. Reproductive states were thus known for all years within an individual's MinLifeWin, such that we did not need to include a detection parameter in our models.

A Bayesian approach was used for inference and implemented in the software program OpenBUGS (Lunn et al. 2009). Markov chain Monte Carlo (MCMC) methods were used to sample, and thus approximate, the posterior distributions of the parameters of interest. The year and individual varying parameters *η*_*t*_, *α*_*i*_, and *β*_*i*_ were modeled hierarchically following independent normal distributions with mean 0 and model- and parameter-specific standard deviations: *η*_*t*_ ∼ *N*(0, *σ*_*η*_), *α*_*i*_ ∼ *N*(0, *σ*_*α*_), and *β*_*i*_ ∼ *N*(0, *σ*_*β*_). Standard deviations *σ*_*α*_ and *σ*_*β*_ are measures of the magnitude of interindividual variability in reproductive rates, for their respective environmental condition, and are thus of primary interest to our question. We chose to model the two individual effects (*α*_*i*_, and *β*_*i*_) as independent between the two time periods, rather than assume a common correlation applied to all individuals by explicitly including it as a parameter of a multivariate normal distribution. If explicitly modeled, the magnitude of the estimated correlation is driven by the individuals behaving similarly in iceberg and noniceberg years, and does not represent the contrasted individual reproductive patterns seen for some individuals. This specification allowed model H3 to be as distinct as possible from the fixed heterogeneity hypothesis represented in model H2. We investigated and quantified correlation under this independence assumption by calculating a correlation coefficient (*ρ*_*α*, *β*_) directly from the joint posterior distribution of the *α*_*i*_'s and *β*_*i*_'s.

A prior for μ was specified through a uniform U(0,1) distribution on the mean of *ψ* on the original scale (i.e., 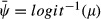). Parameters 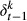, *γ*_1_, and *γ*_2_ were assigned diffuse normal prior distributions N(0,1000) on the logit scale. Uniform prior distributions U(0,10) were used for hyperparameters *σ*_*ε*_, *σ*_*α*_, and *σ*_*β*_. To assess the sensitivity of inferences of the standard deviation of individual effects (*σ*_*α*_) to the choice of prior, we compared these results to those obtained using an inversegamma (4,0.05) prior on variance 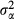 (see Supporting Information). This latter prior distribution has a very high density in values close to zero and thus strongly penalizes high values of variance. This prior therefore penalizes, a priori, the heterogeneity hypothesis (H2), which is a way to assess the support for this hypothesis in a conservative way.

For each of the three competing models, we ran two chains in parallel with different sets of initial values. The first 5000 MCMC samples were discarded (burn-in period), after having checked that convergence was satisfactory. Convergence was visually assessed using sample path plots in conjunction with the Brooks–Gelman–Rubin diagnostic “R” (Brooks and Gelman 1998), with values close to 1.00 indicating adequate convergence. A total of 150,000 MCMC samples after burn-in were used for inference. All parameters described above were defined on the logit scale in the models, but summaries of the posterior distributions provided later in the text, figures, and tables were transformed back to the scale of a probability of reproduction (i.e., interval [0,1]) to ease interpretation. Back-transformed values are hereafter denoted with a star (e.g., 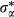).

### Model comparison and hypothesis selection

In addition to making inference directly from parameter posterior distributions, we adopted a model comparison approach. In ecology, the evaluation and comparison of competing models, generally analyzed under a likelihood approach, has traditionally been based on generic criteria of model accuracy (Burnham and Anderson 2002), dominated by the use of the Akaike Information Criterion (AIC). In Bayesian statistics, there currently is no consensus on the best way to select and compare competing models (Link and Barker 2010). Generic criteria, such as AIC, Bayesian Information Criterion, and Deviance Information Criterion are used (Spiegelhalter et al. 2002; Barnett et al. 2010; Cubaynes et al. 2012), but are also widely criticized, especially in the context of hierarchical (random effects) models (Link and Barker 2010). Here, we chose to implement posterior predictive checking to compare the performance of our three competing models (Gelman et al. 2004; Schofield and Barker 2011). The principle of posterior predictive checking (see Gelman et al. 2004, p. 159) is straightforward: if a given model represents a good approximation of the true process that generated the data, then replicated data generated under this model should have very similar features to the observed data. This approach allows assessment of the goodness of fit of each model and provides an explicit tool for model comparison.

For each particular model, the implementation of posterior predictive checking took place as follows. First, 10,000 replicate data sets (*y*^*rep*^) were simulated under different draws from the joint posterior distribution of all parameters to account for uncertainty. As in the statistical models used to analyze the data (see above), the simulation of each individual's reproductive history (i) was conditional on its first and last encounters (i.e., its MinLifeWin was fixed) and (ii) started with state F. Subsequent states were simulated using year- and individual-specific reproductive rates calculated from the set of parameters relevant to each model, thus including the effects of state, age, and year for all models. Second, a relevant function of the data (*T*(·)) was derived for each replicate (*T*(*y*^*rep*^)), and the distribution of *T*(*y*^*rep*^), called the posterior predictive distribution, was compared with the observed value *T*(*y*^*obs*^) derived from the observed data set (*y*^*obs*^). One-sided posterior predictive *P*-values (i.e., *Pr*[*T*(*y*^*rep*^) ≥ *T*(*y*^*obs*^)] or *Pr*[*T*(*y*^*rep*^ ≤ *T*(*y*^*obs*^)]) were then calculated as a summary statistic of the lack of fit between replicated and observed data.

We chose to derive and compare data features directly related to our question of interest. Notably, it has been emphasized in the recent literature that the existence of underlying individual heterogeneity should not be claimed if the observed level of variation in individual performance (e.g., lifetime reproductive success or temporal persistence in a given reproductive state) is not larger than that simply predicted by random chance alone (Tuljapurkar et al. 2009). We therefore decided to explicitly compare the competing models in predicting the observed level of realized interindividual variation in (i) reproductive success and (ii) measures of temporal persistence in the experienced breeder state (E). As a measure of individual reproductive success, we used the observed reproductive output (RepOutput), that is, the number of pups produced by an individual within its MinLifeWin. The measures of individual persistence in the breeder state were defined as follows: (i) the longest time of persistence in the reproductive state (PersistRep), defined as the maximum number of consecutive years an individual remained in state E without skipping a year of reproduction (i.e., length of the longest series of E's for each individual); and (ii) the number of consecutive reproductive events (ConsecRep), defined as the total number of 2-year sequences “EE” in an individual's history. These measures of reproductive performance were calculated for each individual and the level of variability across individuals was investigated through two statistics of interest: (i) the standard deviation (SD) and (ii) the maximum value (Max). To summarize, we thus investigated the SD and Max of the distribution of these three variables (RepOutput, PersistRep, and ConsecRep) over all individuals for each posterior replicate of simulated data (i.e., 10,000 SD's and Max's).

Within each individual's reproductive history, the number of stochastic state transitions is a direct function of the MinLifeWin of this particular individual. Therefore, the level of discrepancy between simulated and observed data is better informed by individuals with long MinLifeWin. To better expose potential lack of fit of each model, the comparison between *T*(*y*^*rep*^) and *T*(*y*^*obs*^) was thus based on the 645 individuals (of a total of 954) having a MinLifeWin of at least 5 years, which we considered a reasonable compromise between sample size and amount of informative data from each individual. Nevertheless, we note that comparisons from samples defined by a different MinLifeWin threshold (e.g., including all individuals) gave similar results and led to the same conclusions.

### Simulations of expected reproductive output

To further investigate the biological meaning of the estimated level of latent individual heterogeneity, we predicted the expected number of descendants, using simulations of individual reproductive trajectories, for three hypothetical types of individuals: (i) a “frail individual” 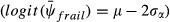, (ii) an “average individual” 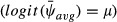, and (iii) a “robust individual” 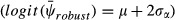. In each individual trajectory, a female started as an 8-year-old first-time breeder (the mean age at first reproduction being 7.62 years in the data) and was allowed to live 10 additional years, which represents the expected future life span for an 8-year-old female. The projections consisted of simulating the stochastic sequence of reproductive states over this fixed time frame, based on posterior means obtained from the best heterogeneity model (H2 or H3) with random year effects drawn [*η*_*t*_ ∼ *N*(0, *σ*_*η*_)] at each time step and applied to the three types of individuals such that they were exposed to the same annual conditions. Results are based on 5000 simulated trajectories for each type of individual.

## Results

Encounter histories from 954 females provided a total of 6792 known-state observations (i.e., “individual-years”), with 954 observations in state F, 3667 in state E, and 2171 in state S. In this data set, individual MinLifeWin (i.e., number of informative years of data, from first reproduction to last sighting) ranged from 2 to 25 years, with an average of 7.1 years per female, and more than 67% of individuals had a MinLifeWin of at least 5 years. The number of pups produced by a female (RepOutput) within its MinLifeWin ranged from 1 to 19, with an average of 4.8 and a standard deviation of 3.2. The two measures of reproductive persistence (ConsecRep and PersistRep) ranged from 0 to 16, and, respectively, averaged 2.1 (with SD = 2.5) and 2.7 (with SD = 2.1).

### Parameter posterior distributions

**Figure 2:**
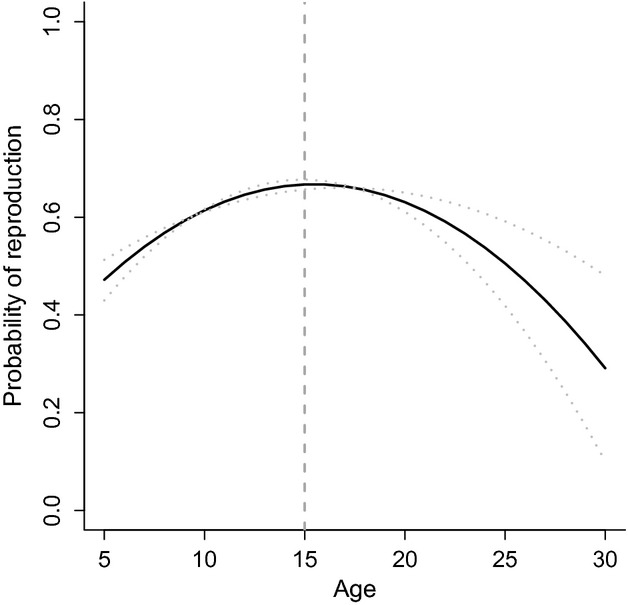
Estimated age-related trend in reproductive rates. The quadratic curve displayed on this graph has been obtained by using posterior means of *γ*_1_ and *γ*_2_ from model H2. The curve obtained by using posterior means of parameters from models H1 and H3 is very similar. The black solid curve represents the mean estimated trend, whereas the gray dotted curves represent the 95% credible interval. The age at which the probability of reproduction is maximum (age 15) is shown by the gray dashed vertical line.

The posterior means of the age parameters (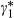 and 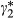) were consistent among the three models (Table 1). The shape of the estimated quadratic trend, imposed in the models, suggests that reproductive rates increased up to age 14–15 and then decreased at older ages (Fig. 2).

**Table 1:** Summary of the posterior distributions of relevant parameters for the three competing models

Parameters	H1. No IH			H2. Fixed IH			H3. Variable IH		
		
	Mean	2.5% LCI	97.5% LCI	Mean	2.5% LCI	97.5% LCI	Mean	2.5% LCI	97.5% LCI
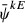	0.62	0.55	0.67	0.66	0.59	0.72	0.65	0.58	0.71
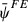	0.50	0.42	0.57	0.54	0.45	0.62	0.52	0.43	0.60
*ψ*^*EE*^	0.67	0.62	0.72	0.67	0.61	0.73	0.67	0.60	0.72
*ψ*^*SE*^	0.67	0.61	0.72	0.76	0.70	0.81	0.74	0.68	0.79
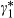	−0.06	−0.10	−0.03	−0.06	−0.10	−0.01	−0.06	−0.10	−0.02
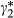	−0.08	−0.12	−0.04	−0.10	−0.15	−0.06	−0.09	−0.14	−0.05
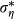	0.12	0.09	0.18	0.14	0.10	0.19	0.13	0.09	0.19
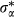	–	–	–	0.15	0.13	0.18	0.13	0.10	0.16
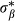	–	–	–	–	–	–	0.18	0.13	0.23
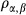	–	–	–	–	–	–	0.11	0.01	0.20

The acronym “IH” stands for “individual heterogeneity”. The mean, and the lower (2.5%) and upper (97.5%) limits of a 95% credible interval (LCI), of the posterior distribution are shown. Symbols with a star (e.g., 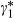) correspond to parameters which value has been transformed back to the more interpretable scale of a probability of reproduction (i.e., in the interval [0,1]). The parameters displayed in this table are as follows: (i) 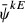, mean reproductive rate, corresponding to a theoretical value (*logit*^−1^(*μ*)) averaging across states (*k*), ages, years, and individuals; (ii) rates of reproduction, specific to the reproductive state *k* at *t* − 1 and averaged across ages, years, and individuals: first-time breeders (*ψ*^*FE*^), experienced breeders (*ψ*^*EE*^), and skip breeders (*ψ*^*SE*^), (iii) first-order 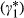 and second-order 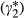 age-effect parameters; we note that both posterior means of 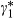 and 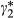 are negative, but the quadratic trend is still parabolic concave (Fig. 2) because age values were standardized (see Methods); (iv) standard deviation of the normal distribution of random year effects 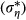, of random “baseline” individual effects 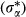, and of random individual effects expressed during iceberg years 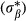; and (v) the correlation between the two types of individual effects (*ρ*_*α*,*β*_).

**Figure 3:**
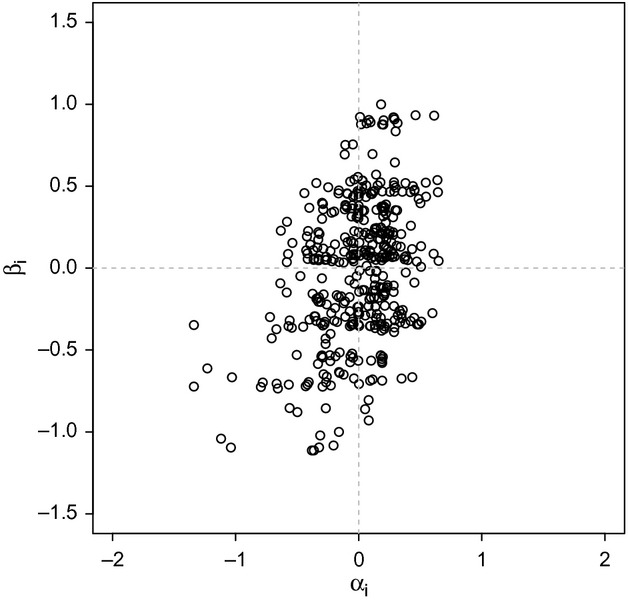
Relationship between the posterior means of individual effects in “normal years” (*α*_*i*_) and in “iceberg years” (*β*_*i*_). The overall relationship appears to be positive, but relatively weak. However, we can clearly note the lack of points in “extreme values” of the lower-right and upper-left corners, indicating that no individuals with a high *α*_*i*_ value have a very low *β*_*i*_ value, and no individuals with a high *β*_*i*_ value have a very low *α*_*i*_ value.

We found clear evidence for the presence of heterogeneity in this population, as the estimated level of “baseline” interindividual standard deviation in reproductive rates 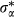 (measured on the interval [0,1]) was distinctly greater than 0 in both model H2 (posterior mean of 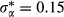, 95% credible interval = [0.13, 0.18]) and model H3 (posterior mean of 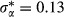, 95% credible interval = [0.10, 0.16], Table 1). It is also interesting to note that the posterior means of 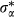 from both models H2 and H3 were of the same order of magnitude as the estimated level of interannual variability 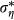 (Table 1). The estimated magnitude of 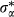 was the same when a restrictive inversegamma prior, penalizing high values, was used (Supporting Information), highlighting the fact that the data provide strong support for this level of underlying individual heterogeneity. In model H3, the posterior mean of 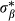 (0.18; 95% credible interval = [0.13, 0.23]) was marginally greater than that of 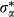, suggesting that interindividual differences might have been amplified during iceberg years. However, the difference between 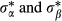 was relatively small (posterior mean of (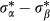) = 0.04, 95% credible interval [−0.01, 0.10]). Hence, we do not make any strong inference about the biological importance of the estimated difference. The estimated correlation between 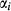 and 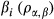 was positive, but relatively weak (posterior mean of 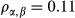, 95% credible interval = [0.01, 0.20], see also Fig. 3), providing suggestive, although inconclusive, evidence of some degree of consistency in individual robustness between normal and iceberg years. For instance, individuals that appeared to be very robust (frail) in normal years were never very frail (robust) in perturbed years (Fig. 3).

Another noteworthy aspect of our results concerns the estimated effect of reproductive state at *t*−1 (Table 1) on current probabilities of reproduction. All models consistently produced posterior means of 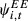 near 0.67 (SD = 0.03), but posterior means of 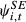 varied by model: model H1 provided a much lower value (about 0.67 [SD = 0.03] and exactly equal to the posterior mean of 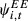) than did models H2 and H3 (about 0.76 [SD = 0.03] and 0.74 [SD = 0.03], respectively). Therefore, when individual heterogeneity was explicitly taken into account (models H2 and H3), we found evidence of a cost of reproduction on subsequent reproductive probability that was not detected when this heterogeneity was ignored. Specifically, models H2 and H3 indicated that a female was more likely to reproduce in a given year if she had skipped reproducing the year before.

### Posterior predictive checks

Overall, the comparison of observed data to simulated data from each model, incorporating uncertainty in all parameters, revealed that the homogeneity model (H1) substantially underpredicted the extent of interindividual differences (measured by SD and Max) in reproductive performance in the observed data, and that better predictions were provided by the fixed individual heterogeneity model (H2) than by the variable individual heterogeneity model (H3).

**Figure 4:**
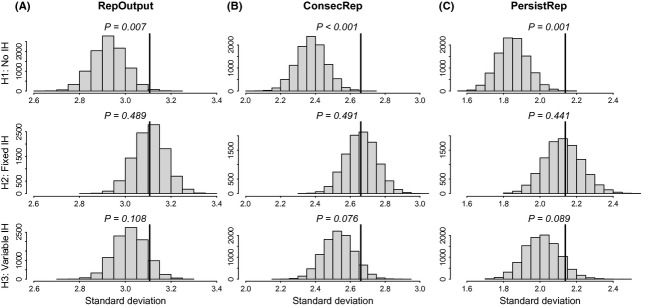
Posterior predictive distributions of the interindividual standard deviation of (A) observed reproductive output (RepOutput), (B) number of transitions from state E to state E (ConsecRep), and (C) maximum persistence in state E (PersistRep), for each model (each row). The standard deviation value from observed data is shown by the vertical black line and the posterior predictive one-sided *P*-value is also displayed above each plot. This posterior predictive analysis was restricted to individuals having a minimum lifetime window of at least 5 years.

**Figure 5:**
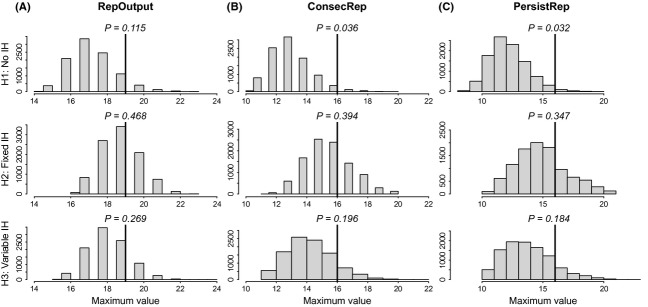
Posterior predictive distribution of the maximum value among all individuals of (A) observed reproductive output (RepOutput), (B) number of transitions from state E to state E (ConsecRep), and (C) maximum persistence in state E (PersistRep), for each model (each row). The maximum value from observed data is shown by the vertical black line and the posterior predictive one-sided *P*-value is also displayed above each plot. This posterior predictive analysis was restricted to individuals having a minimum lifetime window of at least 5 years.

More specifically, we found that individual variability in RepOutput was poorly predicted by model H1: indeed, only 0.7% of the simulated data sets (*y*^*rep*^) obtained from model H1 produced SDs of RepOutput as large as the observed SD (Fig. 4: *P* = 0.007). Model H1 also tended to underpredict the Max value of RepOutput (Fig. 5: *P* = 0.115). The poor performance of model H1 at accounting for the observed level of individual heterogeneity was even more evident when considering measures of reproductive persistence (SD of ConsecRep: *P* < 0.001; SD of PersistRep: *P* = 0.001; and Max of ConsecRep: *P* = 0.036; Max of PersistRep: *P* = 0.032). In contrast, the fixed heterogeneity model (H2) provided predictions that were well distributed around the observed values of all these features (all *P*-values between 0.347 and 0.491). The posterior predictive performance of the variable heterogeneity model (H3) appeared to be intermediate, with a slight underprediction of the SD (Fig. 4), and to a lesser extent, of the Max (Fig. 5) of the different measures of reproductive performance.

These results clearly show that individual heterogeneity in reproductive rates must be accounted for to explain the level of individual variability observed in reproductive performance. Moreover, it seems that this heterogeneity is better represented as a fixed feature than as a variable feature. Finally, when individual heterogeneity in reproductive rates was incorporated, costs of reproduction to future reproduction were detected.

### Simulations of expected reproductive output

**Figure 6:**
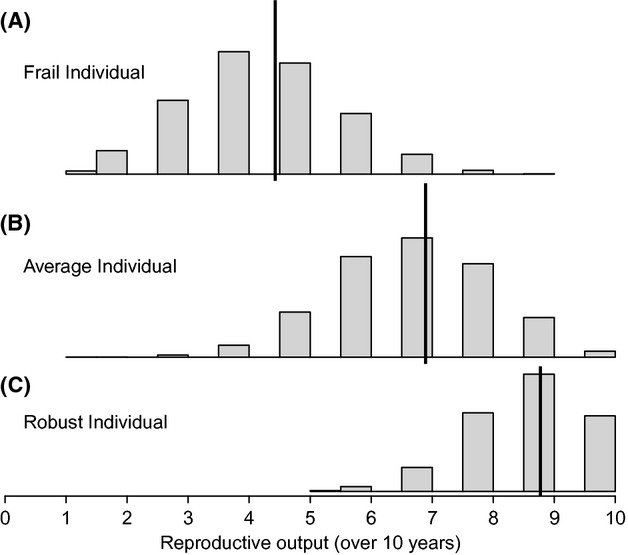
Approximated distributions of the expected reproductive output for three types of individuals: (A) “frail” individual; (B) “average” individual; and (C) “robust” individual (see text for details). These results are based on 5000 simulated trajectories for each type of individual. The mean of each distribution is shown by a black vertical line.

The approximate expected reproductive output (±SD) was 4.4 (±1.3) pups for “frail” females, 6.9 (±1.3) pups for “average” females, and 8.8 (±1.0) pups for “robust” females (Fig. 6). A “robust” individual is thus expected to produce on average twice as many pups as a “frail” individual over a 10-year period, a result that strongly suggests that the estimated level of individual heterogeneity in reproductive probabilities translates into substantial differences in terms of expected reproductive output.

## Discussion

We investigated three competing hypotheses concerned with the existence and the temporal expression of individual heterogeneity in reproductive rates of females in a population of Weddell seals. Results provided strong support for the presence of latent individual heterogeneity in reproduction and the expression of this heterogeneity seemed relatively independent from environmental conditions. We also detected a substantial cost of reproduction when individual heterogeneity was explicitly accounted for in the models, which is a novel finding for this species and has important implications for future studies of reproductive costs in other species.

### Strong evidence for the presence of latent individual heterogeneity

Overall, we found conclusive evidence for the existence of latent individual heterogeneity in reproductive rates. The estimated magnitude of interindividual variability 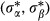 from models H2 and H3 was high and clearly distinct from zero, even when a penalizing prior was used (Supporting Information). Moreover, heterogeneity models, especially model H2, provided predictions of realized among-individual variability in reproductive performances that were similar to the observed levels, whereas estimators ignoring underlying individual heterogeneity (from model H1) tended to substantially underpredict the degree of realized among-individual variability. This latter result reveals that the magnitude of “residual” (i.e., after having accounted for age, year, and breeding state) interindividual variability observed in the population cannot be adequately explained as the simple result of a chance mechanism (stochastic nature of individual trajectories; Tuljapurkar et al. 2009). These findings provide good support for the existence of latent individual heterogeneity in this population. Although the sources of this individual variation in reproductive performance remain unidentified so far, a relevant speculation is that individual seals probably differ in their ability to acquire, store, and conserve energy resources (Van Noordwijk and De Jong 1986). In Weddell seals, which mainly feed on fish and squids (Burns et al. 1998), individual differences in resource acquisition might be directly linked to physical, physiological, or behavioral attributes that determine diving and hunting abilities (Costa et al. 2004; Williams et al. 2004). Different individuals might also adopt different strategies regarding the timing, duration, and location of foraging bouts (K. Goetz and D. Costa pers. comm.; A. Hindle, J.-A. Mellish, and M. Horning pers. comm.). Given the probable spatial and temporal variability in food availability and competitor abundance in the Ross Sea (Guglielmo et al. 1998, 2009; Saggiomo et al. 1998; Smith et al. 2007), different strategies could have an influence on the foraging efficiency of individual seals. On the other hand, differences might also be linked to physiological and metabolic functions that influence the efficiency of storage, consumption, and/or conservation of energy resources (Kooyman et al. 1973; Castellini et al. 1992; Wheatley et al. 2006, 2008a,b). Furthermore, differences might exist in phenotypic attributes that directly affect fecundity, such as oocyte quality or success of embryo implantation (Atkinson 1997). We, however, hypothesize that attributes affecting the efficiency of acquisition and conservation of energy resources play a much more important role in determining the observed level of individual heterogeneity, especially as female Weddell seals are essentially capital breeders for which reproductive success heavily depends on body fat reserves (Boness and Bowen 1996; Wheatley et al. 2008a,b).

The estimated extent of among-individual variability appeared to be of the same order of magnitude as the estimated level of interannual variability, which is known to be a major component of vital rate variation in this population (Hadley et al. 2007b; Rotella et al. 2012; Chambert et al. 2012). Interindividual differences are thus responsible for a non-negligible amount of variation in reproductive rates. This novel result reveals that individual heterogeneity should not be ignored (Weladji et al. 2008; Gimenez and Choquet 2010; Hamel et al. 2012b) in modeling exercises aiming at capturing the major sources of vital rate variation in this population. Our simulation work also reveals that the amount of reproductive individual heterogeneity found in this population translates into substantial differences in terms of expected reproductive output. Although reproductive output is only one of the several components of true fitness, this result suggests that fitness might vary quite substantially among individuals of this population. Differences in the survival and future reproductive success of offspring will, however, need to be investigated in order to obtain a more thorough measure of individual heterogeneity in fitness. Producing more offspring is not necessarily a better strategy, especially in species with high parental care investment, as a female might have to trade-off the quality (i.e., less maternal investment) of each offspring to produce more of them. On the other hand, if maternal investment is relatively homogeneous, then the fate of any offspring is relatively independent from its particular mother, and therefore, a female that produces more offspring will have a higher expected fitness. Overall, this is an important result of this study because the quantification of fitness variation in natural populations (Link et al. 2002) is a crucial step to better understand broad evolutionary processes. At present, there are few examples of such direct quantification of individual variation in fitness components of animals in the wild, and they remain limited to a small number of highly detectable species (e.g., *Rissa tridactyla*: Cam et al. 2002; *Marmota flaviventris*: Oli & Armitage 2003).

### Marginal evidence for a temporally variable expression of latent individual heterogeneity

From the results of model H3, we were able to investigate whether, during “perturbed” environmental conditions, (i) the hierarchy in robustness among individuals would change and (ii) the magnitude of individual heterogeneity would be amplified. First, we found mild support for our prediction that the hierarchy among individuals would be maintained across the two contrasted environmental conditions. Indeed, the posterior mean correlation between individual effects in the two contrasted conditions was positive, as predicted, but still relatively low (*ρ*_*α*, *β*_ = 0.11). Nevertheless, even though the relative hierarchy certainly changed among many individuals, there was still some consistency in the robustness, as no individuals went from one extreme to the other (Fig. 3). This result suggests that the most robust individuals in normal years tend to also be more robust than average in perturbed years (and vice versa), in support with the fixed heterogeneity hypothesis (Cam et al. 2002). Concerning the potential amplification of the magnitude of individual heterogeneity in perturbed conditions, we also found only suggestive evidence for the predicted pattern, as the estimate of interindividual variability appeared to be only marginally larger in the perturbed period than in normal years. In spite of the inconclusive evidence provided by these data, we note that the results were in the predicted direction, suggesting that interindividual differences might be more apparent when environmental conditions are harsh. This trend is in accordance with theoretical predictions (Lomnicki 1978) and empirical results from previous work on seabirds (Barbraud and Weimerskirch 2005) and ungulates (Tavecchia et al. 2005).

On the other hand, the posterior predictive performance of model H3 appeared to be poorer than that of the simpler model H2, a result that highlights the relevance of the “fixed heterogeneity” hypothesis for representing the key aspect of the individual heterogeneity that prevails in the study population. As other studies have suggested that interactions between individual heterogeneity and environmental conditions might be relatively common in nature (Lomnicki 1978; Hamel et al. 2009a,b), we can wonder why we might have failed to find clear evidence of such an interaction here. This might be due to the fact that the 5-year iceberg event (MacAyeal et al. 2008) used here as a proxy of perturbed environmental conditions might have been too brief to allow a precise assessment of how it affected the expression of individual heterogeneity. Indeed, even though the investigation of such unique large-scale perturbations can be very informative (Carpenter 1990), the detection of complex interactive effects, such as the ones predicted here, might require longer periods of contrasted environments. Overall, we therefore conclude that, because our data could not strongly support hypothesis H3, the “fixed heterogeneity” hypothesis (H2) remains the best representation of the individual heterogeneity in reproductive probabilities existing in this population.

### Evidence of a cost of reproduction

In these analyses, we also shed light on the relatively important cost of current reproduction on future reproduction, and revealed that this is only detected when individual heterogeneity is accounted for in the model (Vaupel and Yashin 1985; Weladji et al. 2008). In a previous study of the same population, Hadley et al. (2007a) found no evidence for such a reproductive cost in recruited individuals, and even observed a pattern opposite to what had been expected as the estimated probability of reproduction in year *t* was slightly lower for individuals having skipped reproducing the previous year 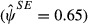 than for those having bred the previous year 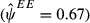. In this study, posterior means similar to the latter estimates were obtained from model H1 (Table 1), but posterior means from models H2 and H3 provided clear evidence of a productive cost (e.g., model H2: *ψ*^*SE*^ = 0.76 vs. *ψ*^*EE*^ = 0.67). Models ignoring individual heterogeneity failed to detect the difference between *ψ*^*SE*^ and *ψ*^*EE*^ because these state-specific estimates were averaged over all types of individuals. As frail females skip reproduction more often than other females, they provide more data to transitions from state S (either toward state E, with probability *ψ*^*SE*^, or toward state S, with probability *ψ*^*SS*^ = 1 − *ψ*^*SE*^) and thus have a greater influence on the estimation of *ψ*^*SE*^, and because frail females have lower reproductive rates (more transitions “S to S” than average), they tend to pull the averaged value of *ψ*^*SE*^ down. Including individual heterogeneity in the model disentangles the influence of reproductive state at *t*−1 (e.g., influence of energy expenditure/allocation), which is common to all females, from the influence of individual robustness, which is specific to each female. The magnitude of the estimated cost is such that, on average, when a female reproduces in a given year, she is about 10% less likely to breed the next year than if she had skipped reproduction. This is one of the first evidence of such a reproductive cost in a population of long-lived marine mammal living in a polar environment, and it supports findings of previous studies that have shown the necessity of accounting for individual heterogeneity to highlight, in demographic data, these types of life-history trade-offs occurring at the individual scale (Vaupel and Yashin 1985; Van Noordwijk and De Jong 1986; Service 2000; Cam et al. 2002; Nussey et al. 2008; Weladji et al. 2008).

### Conclusion and perspectives

The growing number of demographic studies using individual-level models and explicitly investigating individual heterogeneity will certainly allow improvement in our understanding of the prevalence, the magnitude, and the nature of interindividual differences in fitness components, as well as in our comprehension of the expression of life-history trade-offs in wild populations (Service 2000; Weladji et al. 2008). Furthermore, given that individual heterogeneity can have an influence on demographic stochasticity (Kendall et al. 2011), the accounting of individual effects in population dynamics models should enhance the accuracy of demographic projections, especially for small and threatened populations.

## Supporting Information

**Data S1.** Estimation of the magnitude of the variance of individual random effects with a restrictive and penalizing prior.

**Figure S1.** Histograms representing the probability density of the variance () for the two different priors used for model H2: (a) & (c) uniform U(0,10) distribution on the corresponding standard deviation *σ*_*α*_; (b) & (d) inversegamma (4,0.05) distribution on . For better readability, we show the densities on two different x-axis scales: histograms (a) and (b) are scaled on [0;0.15], where the density of  values under the inversegamma prior (b) can more easily be interpreted; histograms (c) and (d) are scaled on [0;100], where the full extent of the density of  values under the uniform prior (c) can be better seen. We see that the density of  values is heavily shifted toward very small values in the case of the inversegamma (4,0.05) prior distribution compared with the case of the uniform U(0,10) prior distribution. On the [0;100] scale, the density of the inversegamma (4,0.05) distribution looks as a single peak at zero.

**Table S1.** Summary of the parameter posterior distributions from model H2 (“fixed heterogeneity” hypothesis; see main text) using two contrasted priors on the variance () of random individual effects: (i) the inversegamma (4,0.05) distribution on  which puts a lot of weight on very small values (close to zero), and thus strongly penalizes high values; (ii) the uniform U(0,10) distribution on the corresponding standard deviation *σ*_*α*_, which is a very vague prior and has more weight on high values of variance. The mean, the standard deviation (SD), 2.5% and 97.5% percentiles (perc.) of the posterior distribution are shown. Symbols with a star (e.g., ) correspond to parameters back-transformed to the more interpretable scale of a probability of reproduction (i.e., in the interval [0,1]). See main text for more details.
