# Supplementary material for: Individual heterogeneity in reproductive rates and cost of reproduction in a long-lived vertebrate
Source: Ecol Evol. 2013 May 31;3(7):2047–60. doi: 10.1002/ece3.615 (PMC3728946; doi:10.1002/ece3.615)
Supplement: Supplementary file 1 [file ece30003-2047-SD1.docx]

**Supporting Information**

**Estimation of the magnitude of the variance of individual random effects with a restrictive and penalizing prior**

Here, we present the results from model H2 (see main text, Material and Methods - *Statistical modeling*) obtained when we used an inverse-gamma(4, 0.05) prior distribution for the variance of $\alpha_{i}$ ($\sigma_{\alpha}^{2}$) instead of the uniform prior U(0,10) on the standard deviation $\sigma_{\alpha}$ that was used to obtain the results presented in the main mauscript. Although the U(0,10) distribution was used as a weakly informative prior, the inverse-gamma(4, 0.05) prior used here strongly penalizes high values of $\sigma_{\alpha}^{2}$, and therefore, allows a more stringent assessment of the degree of support of the ‘fixed heterogeneity’ hypothesis (H2). This comparison aimed to assess the sensitivity of our estimates of $\sigma_{\alpha}$ to the choice of its prior, thus evaluating the strength of our inference regarding the magnitude of this parameter.

The characteristics of the inverse-gamma(4, 0.05) distribution are the following: the mean of the distribution is 0.017, the mode is 0.010 and the standard deviation is about 0.012. In terms of quantiles, 95% of the values are below 0.037, and 99% are below 0.061 (see Figure S1). The integrated probability of values greater than the H2 posterior mean, 0.462, is 7.e-06. These properties clearly illustrate that this inverse-gamma prior distribution strongly penalizes high variance values, and therefore, the ‘fixed heterogeneity’ hypothesis H2.

We ran 2 parallel MCMCs of 200,000 iterations, after a burn-in period of 5,000 iterations, to sample the posterior distribution of model parameters. The convergence of the MCMC chains was checked in the same way as were other models (see main text, Material and Methods).

The posterior distributions of all parameters were virtually identical to those obtained from the original model H2 using a U(0,10) prior (Table S1). Using the IG(4,0.05) prior, the posterior mean of the standard deviation of $\alpha_{i}$, on original scale, is 0.14 (posterior SD = 0.01; 95% credible interval: [0.12, 0.17]) , compared to 0.15 (posterior SD = 0.01; 95% credible interval [0.13, 0.18]) for the original model using a U(0,10) prior. This comparison demonstrates the amount of information in the data was able to overcome the penalizing prior. This result strengthens our conclusions regarding the relatively high magnitude of among-individual variability in reproductive rates and thus the relevance of the ‘heterogeneity hypothesis’ H2 versus the homogeneity hypothesis H1.

**Figure S1.** Histograms representing the probability density of the variance ($\sigma_{\alpha}^{2}$) for the two different priors used for model H2: (a) & (c) uniform U(0,10) distribution on the corresponding standard deviation $\sigma_{\alpha}$; (b) & (d) inverse-gamma(4,0.05) distribution on $\sigma_{\alpha}^{2}$. For better readability, we show the densities on two different x-axis scales: histograms (a) and (b) are scaled on [0;0.15], where the density of $\sigma_{\alpha}^{2}$values under the inverse-gamma prior (b) can more easily be interpreted; histograms (c) and (d) are scaled on [0;100], where the full extent of the density of $\sigma_{\alpha}^{2}$values under the uniform prior (c) can be better seen. We see that the density of $\sigma_{\alpha}^{2}$values is heavily shifted towards very small values in the case inverse-gamma(4,0.05) prior distribution compared to the case of the uniform U(0,10) prior distribution. On the [0;100] scale, the density of the inverse-gamma(4;0.05) distribution looks as a single peak at zero.


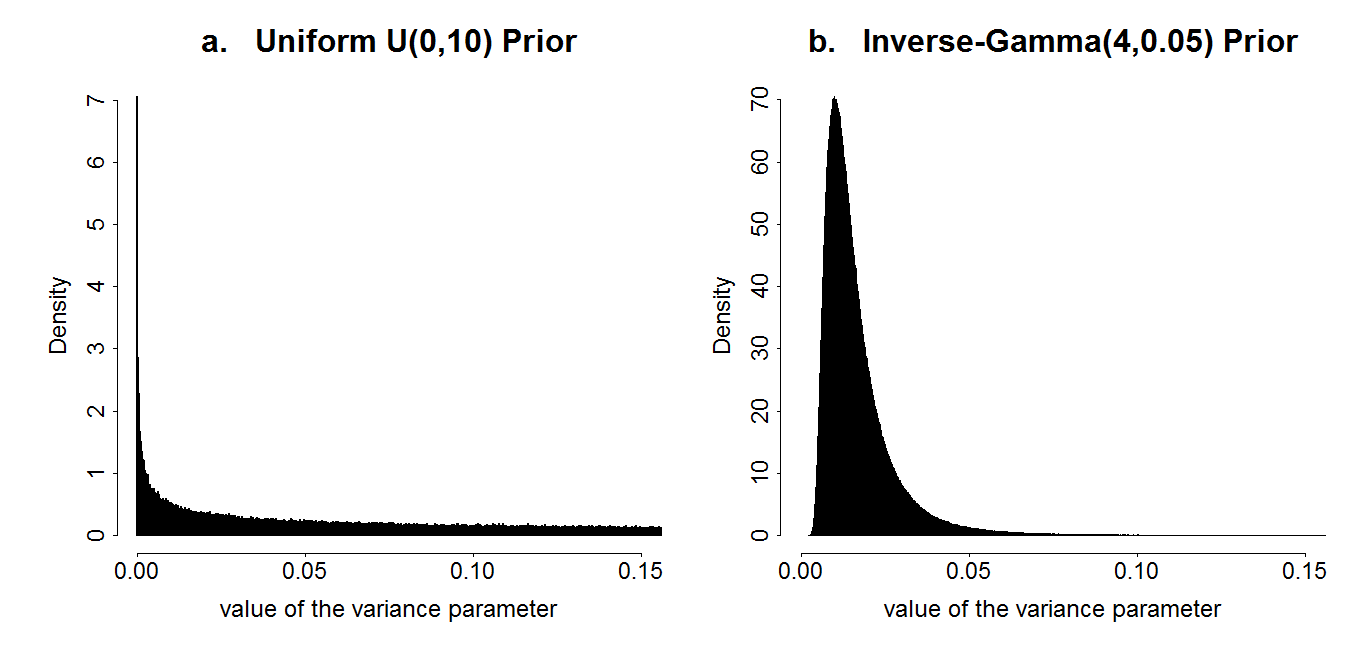

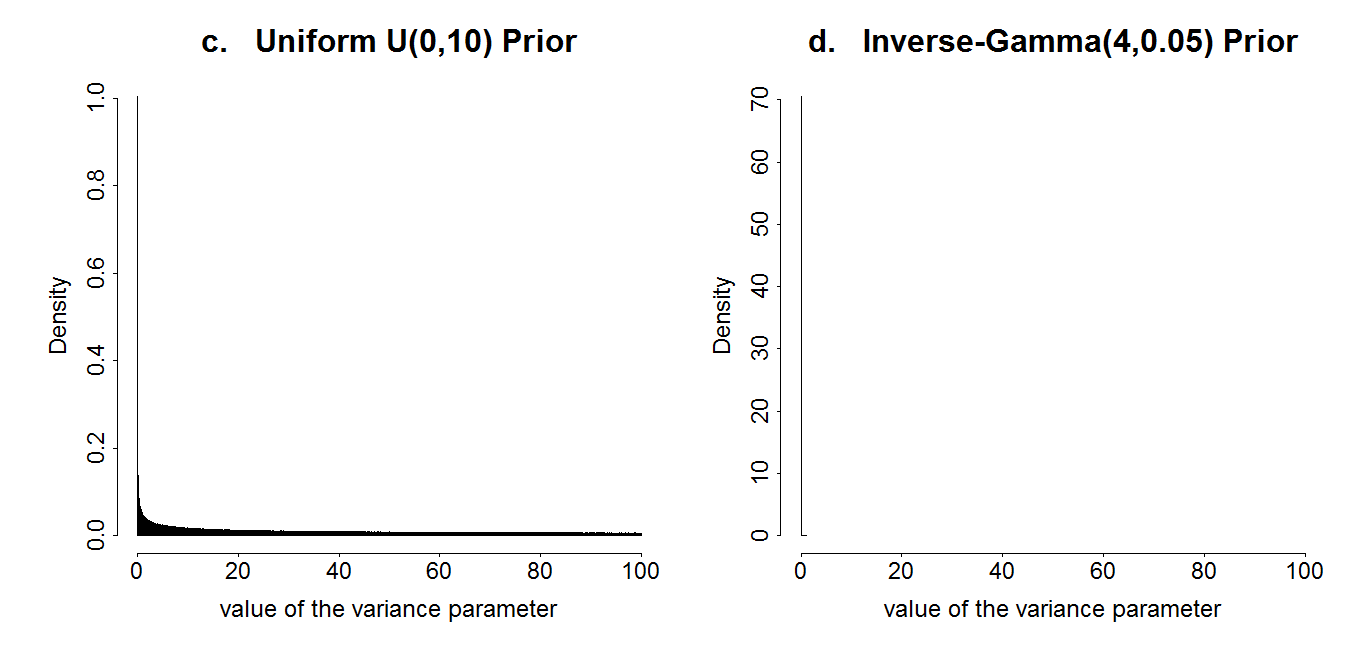


**Table S1**. Summary of the parameter posterior distributions from model H2 (‘fixed heterogeneity’ hypothesis; see main text) using two contrasted priors on the variance ($\sigma_{\alpha}^{2}$) of random individual effects: (i) the inverse-gamma(4,0.05) distribution on $\sigma_{\alpha}^{2}$which puts a lot of weight on very small values (close to zero), and thus strongly penalizes high values; (ii) the uniform U(0,10) distribution on the corresponding standard deviation $\sigma_{\alpha}$, which is a very vague prior and has more weight on high values of variance. The mean, the standard deviation (SD), 2.5% and 97.5% percentiles (perc.) of the posterior distribution are shown. Symbols with a star (e.g., $\gamma_{1}^{*}$) correspond to parameters back-transformed to the more interpretable scale of a probability of reproduction (i.e., in the interval [0,1]). See main text for more details.

| Parameters | **MODEL H2 with Inverse-Gamma prior** | | | |  | **MODEL H2 with Uniform prior** | | | |
| --- | --- | --- | --- | --- | --- | --- | --- | --- | --- |
|  | Mean | SD | 2.5% perc. | 97.5% perc. |  | Mean | SD | 2.5% perc. | 97.5% perc. |
| $\bar{\psi}^{kE}$ | 0.66 | 0.03 | 0.59 | 0.72 |  | 0.66 | 0.03 | 0.59 | 0.72 |
| $\psi^{FE}$ | 0.53 | 0.04 | 0.45 | 0.62 |  | 0.54 | 0.04 | 0.45 | 0.62 |
| $\psi^{EE}$ | 0.67 | 0.03 | 0.60 | 0.73 |  | 0.67 | 0.03 | 0.61 | 0.73 |
| $\psi^{SE}$ | 0.75 | 0.03 | 0.69 | 0.80 |  | 0.76 | 0.03 | 0.70 | 0.81 |
| $\gamma_{1}^{*}$ | -0.06 | 0.02 | -0.10 | -0.01 |  | -0.06 | 0.02 | -0.10 | -0.01 |
| $\gamma_{2}^{*}$ | -0.10 | 0.02 | -0.15 | -0.06 |  | -0.10 | 0.02 | -0.15 | -0.06 |
| $\sigma_{\eta}^{*}$ | 0.13 | 0.02 | 0.10 | 0.19 |  | 0.14 | 0.03 | 0.10 | 0.19 |
| $\sigma_{\alpha}^{*}$ | 0.14 | 0.01 | 0.12 | 0.17 |  | 0.15 | 0.01 | 0.13 | 0.18 |
